# A study on predictive factors and mediational pathways of depression among college students in China based on structural equation modeling

**DOI:** 10.3389/fpubh.2026.1758715

**Published:** 2026-02-26

**Authors:** Xiuli Qing, Le Yang

**Affiliations:** 1 Department of Obstetrics Nursing, West China Second University Hospital, Sichuan University, Chengdu, China; 2 Key Laboratory of Birth and Related Diseases of Women and Children (Sichuan University), Ministry of Education, Chengdu, China; 3 School of Marxism, Sichuan University, Chengdu, China

**Keywords:** childhood trauma, college students, depression, internet addiction, perceived stress, self-esteem, structural equation modeling

## Abstract

**Objective:**

This study aimed to explore the key predictive factors and underlying theorized mediating pathways of depression among Chinese college students using structural equation modeling (SEM), with the goal of providing empirical evidence to support mental health interventions in higher education.

**Methods:**

A longitudinal survey was conducted among undergraduate students from a comprehensive university in Sichuan Province, China. Data were collected at two time points: at enrollment (T1, September–October 2022) and three months later (T2, December 2022–January 2023). Participants were divided into depression and non-depression groups based on Self-Rating Depression Scale (SDS) scores. Between-group differences were analyzed using chi-square, t-tests, or Wilcoxon rank-sum tests. SEM was employed to examine direct and indirect pathways among childhood trauma, self-esteem, coping styles, internet addiction, perceived stress, and depression.

**Results:**

A total of 880 valid samples were included, with 154 (17.50%) assigned to the depression group. Univariate analysis indicated that sociodemographic factors, family environment, coping style, and internet behavior were significantly associated with depression. SEM results revealed that childhood trauma positively influenced depression both directly and through multiple mediators, including reduced self-esteem, increased negative coping, internet addiction, and elevated perceived stress. Self-esteem and negative coping also exhibited dual pathways affecting depression, with perceived stress serving as a critical mediator.

**Conclusion:**

Depression among college students is influenced by the interaction of early experiences, psychological traits, and behavioral factors. A multi-level intervention strategy-ranging from risk screening and stress management to trauma-informed support-is recommended for university mental health services.

## 1 Introduction

Depression is a common and severe mental disorder characterized by persistent low mood, loss of interest, feelings of worthlessness, attention deficits, and in severe cases, suicidal ideation (1–3). Globally, more than 280 million people are affected by depression (4), with China accounting for approximately 95 million cases (5, 6). Previous studies suggest that college students are particularly vulnerable, showing higher prevalence rates (24–34%) compared to the general population (7–9). Depression not only impairs academic performance and social functioning but may also have long-term effects on career development and quality of life.

Although numerous studies have investigated depression among college students, several limitations remain. Many studies rely on cross-sectional designs, which hinder causal inference (>85% of published works). Sample representativeness is often limited, with overrepresentation of certain majors such as psychology (10–15). Furthermore, most studies adopt a single theoretical perspective, leading to incomplete variable selection and an inability to capture the complex mediating theorized mediating pathways involved.

Recent research has begun to explore the role of modern behavioral factors such as internet addiction in depression. Neuroimaging studies suggest that excessive internet use may lead to prefrontal cortex dysfunction and disrupted sleep rhythms (16, 17). Cognitive-behavioral models further propose that internet addiction reinforces negative cognitive schemas, thereby exacerbating depressive symptoms (18). However, systematic empirical evidence regarding the mediating role of internet addiction in the context of other established risk factors—such as childhood trauma, self-esteem, and coping styles—is still limited.

Building upon the aforementioned research status and limitations, this study integrates the Diathesis-Stress Model with a developmental psychopathology perspective to construct a multidimensional theoretical framework for systematically investigating the formation mechanisms of depression among Chinese college students. This framework conceptualizes early negative experiences (such as abuse and neglect) as significant diathesis factors, which shape individuals’ relatively stable psychological traits (e.g., low self-esteem) and maladaptive cognitive-behavioral patterns (e.g., negative coping tendencies, propensity for internet addiction). These diathesis factors interact with stress factors encountered during the university adaptation phase (e.g., perceived stress), ultimately influencing the risk of developing depression (18).

Based on this framework, we propose the following core research hypotheses:

1. Childhood abuse will function as a distal risk factor, not only directly predicting depression but also indirectly increasing depression risk by reducing self-esteem levels, enhancing negative coping tendencies, and promoting internet addiction behaviors.
2. Self-esteem and negative coping styles will not only directly predict depressive symptoms but will also exert their influence by mediating the individual’s level of perceived stress.
3. Perceived stress plays a central proximal mediating role during the university adaptation period, serving as a key bridge connecting early diathesis factors, psychological-behavioral factors, and depressive symptoms.

Therefore, this study aims, through a prospective survey of Chinese freshmen employing Structural Equation Modeling (SEM), to test the theoretical framework outlined above. It seeks to elucidate the direct and indirect pathways through which factors such as childhood abuse, self-esteem, coping styles, internet addiction, and perceived stress affect depression among college students, thereby clarifying their underlying mechanisms.

## 2 Methods

### 2.1 Research design

A two-stage longitudinal survey was conducted at a university in Sichuan Province, China. Freshmen were recruited in September–October 2022 (T1). Using stratified random sampling, participants were selected from four disciplinary categories: humanities, sciences, engineering, and medicine. Two colleges were randomly chosen from each category, followed by cluster sampling within selected colleges.

Baseline assessment included demographic information, childhood trauma, self-esteem, coping style, internet addiction, perceived social support, parental attachment, negative life events, perceived stress, and depression levels. Follow-up was conducted three months later (T2) to assess depression incidence. Participants with depression at baseline were excluded to control for reverse causality. The study was approved by the university ethics committee, and informed consent was obtained from all participants.

### 2.2 Study population

#### 2.2.1 Sampling method

The sample was drawn from a university in Sichuan Province 2022 undergraduate cohort. Stratified random sampling was used based on four disciplinary categories: humanities, sciences, engineering, and medicine. Two colleges were randomly selected from each category, followed by cluster sampling within the selected colleges.

#### 2.2.2 Inclusion and exclusion criteria

The study aimed to examine the impact of factors measured at enrollment on the incidence of depression measured three months later. Since literature indicates a high correlation (approximately 0.6) between baseline and follow-up depression levels (19, 20), baseline depression cases were excluded to control for confounding.

### 2.3 Sample size calculation

$$n=\frac{u_{\alpha /2}^{2}\pi (1-\pi )}{\delta^{2}}$$

Using the sample size formula above, and based on the prevalence rate of depression among Chinese college students (*π* = 29.3%) (21) with a margin of error (*δ* = 5.2%), the calculated sample size was 295. Additionally, since the survey contained 170 items, and the sample size should be at least 5 times the number of items, the minimum sample size required was 850. Ultimately, 880 participants were included.

### 2.4 Research tools

#### 2.4.1 Self-rating depression scale (SDS)

Assessed depression levels using 20 items. Total scores were multiplied by 1.25 and rounded: <53 indicated no depression, 53–62 mild depression, 63–72 moderate depression, and >73 severe depression (22, 23).

#### 2.4.2 Childhood trauma questionnaire (CTQ)

The Chinese version of CTQ-SF, revised by Zhao et al. (24), measured childhood abuse experiences. Higher scores indicated more severe abuse (24).

#### 2.4.3 Self-esteem scale (SES)

Measured self-esteem levels with 6 forward-scored items and 4 reverse-scored items. Higher total scores indicated higher self-esteem (25).

#### 2.4.4 Trait coping style questionnaire

Assessed coping styles under external stimuli, divided into positive coping (10 items) and negative coping (10 items) dimensions. Total scores were calculated for each dimension (26).

#### 2.4.5 Perceived social support scale (PSSS)

Measured perceived social support from family, friends, and others. Higher scores indicated greater perceived support (3).

#### 2.4.6 Inventory of parent and peer attachment (IPPA)

Assessed attachment levels to parents and peers. This study used the parental attachment subscale, with separate scores for father and mother attachment (10 items each) (27).

#### 2.4.7 Adolescent self-rating life events checklist (ASLEC)

Measured the number and impact of life events. Scores from 5 dimensions were summed to represent total stress load (28).

#### 2.4.8 Chinese perceived stress scale (CPSS-14)

Assessed perceived stress levels, divided into feelings of loss of control and tension (7 items each). Total scores represented overall perceived stress (29).

#### 2.4.9 Young’s internet addiction test (IAT)

Evaluated internet addiction severity with 20 items rated on a 1–5 scale. Total scores up to 100 indicated higher addiction severity (30).

### 2.5 Quality control

Pre-survey average completion time was 24 min. Responses completed in <10 min were considered invalid and excluded. Student ID matching with the university’s database ensured accuracy, excluding incorrect or incomplete entries.

### 2.6 Data analysis

A two-stage integrated analysis of “statistical screening-structural modeling” was employed. Participants were first divided into depression and non-depression groups based on clinical cutoff scores. Between-group differences were analyzed to inform variable selection for SEM.

Categorical variables were compared using chi-square or Fisher’s exact tests. Normally distributed continuous variables were analyzed with independent t-tests, while non-normal data used Wilcoxon rank-sum tests. Significant variables from univariate analysis were incorporated into SEM to systematically analyze pathways influencing depression.

It is crucial to emphasize that the Structural Equation Modeling (SEM) employed in this study is fundamentally a covariance-based associative model. While the prospective design provides a stronger basis for inferring temporal sequence than cross-sectional data, the analysis does not control for all unmeasured confounding factors, nor does it test for potential bidirectional relationships among variables. Therefore, the path coefficients reported in the manuscript should be interpreted as evidence for predictive effects based on temporal precedence and theorized mediating pathways, rather than as confirmed causal relationships.

The SEM parameters were estimated using the maximum likelihood method. To preserve the original units of measurement and directly reflect the impact of a unit change in the predictor variables on the outcome variable, all path coefficients reported in the manuscript, including those in Table 1, are unstandardized coefficients. When comparing the effect sizes of different variables on depression, readers should focus on the coefficient values and their confidence intervals.

**Table 1:** The results of unstandardized path coefficients for the final structural equation model.

Path	Estimate	95% CI	*p*-value
Total effects			
Depression ← Perceived stress	0.57	(0.48, 0.66)	***
Depression ← Self-esteem	−0.88	(−1.01, −0.75)	***
Depression ← Negative coping	0.41	(0.29, 0.50)	0.001
Depression ← Childhood abuse	3.22	(2.34, 4.99)	0.001
Depression ← Internet addiction	0.03	(0.02, 0.06)	0.001
Perceived stress ← Internet addiction	0.06	(0.03, 0.09)	***
Perceived stress ← Negative coping	0.45	(0.38, 0.51)	***
Perceived stress ← Self-esteem	−0.54	(−0.63, −0.46)	***
Perceived stress ← Childhood abuse	2.63	(1.90, 4.05)	0.001
Internet addiction ← Negative coping	0.38	(0.25, 0.50)	***
Internet addiction ← Childhood abuse	3.70	(2.24, 6.26)	***
Negative coping ← Childhood abuse	2.66	(1.94, 3.37)	***
Self-esteem ← Childhood abuse	−2.34	(−2.89, −1.78)	***
Direct effects			
Negative coping ← Childhood abuse	2.66	(1.94, 3.37)	***
Internet addiction ← Negative coping	0.38	(0.25, 0.50)	***
Self-esteem ← Childhood abuse	−2.34	(−2.89, −1.78)	***
Internet Addiction ← Childhood abuse	2.70	(1.57, 3.82)	***
Perceived stress ← Internet addiction	0.06	(0.03, 0.09)	***
Perceived stress ← Negative coping	0.43	(0.37, 0.48)	***
Perceived stress ← Self-esteem	−0.54	(−0.63, −0.46)	***
Depression ← Perceived stress	0.57	(0.48, 0.66)	***
Depression ← Self-esteem	−0.57	(−0.70, −0.45)	***
Depression ← Negative coping	0.15	(0.06, 0.24)	0.001
Indirect effects			
Depression ← Childhood abuse	3.22	(2.34, 4.99)	0.001
Depression ← Self-esteem	−0.31	(−0.39, −0.24)	0.001
Depression ← Negative coping	0.26	(0.20, 0.31)	0.001
Depression ← Internet addiction	0.03	(0.02, 0.06)	0.001
Perceived stress ← Negative coping	0.02	(0.01, 0.04)	0.001
Perceived stress ← Childhood abuse	2.63	(1.90, 4.05)	0.001
Internet addiction ← Childhood abuse	1.00	(0.63, 1.64)	0.0

This table presents the unstandardized path coefficient estimates and their 95% confidence intervals. *** *p* < 0.001.

We acknowledge that conducting multiple between-group comparisons (including t-tests / Wilcoxon rank-sum tests for continuous variables and chi-square tests for categorical variables) between the depression and non-depression groups increases the risk of Type I error. The primary aim of this initial analysis was to comprehensively explore potential factors associated with depressive states, rather than to conduct a confirmatory test of a few specific *a priori* hypotheses. Therefore, the statistical approach prioritized the identification of meaningful effects and associations to inform subsequent variable selection for model building. To balance exploratory analysis with statistical rigor, we did not apply multiple comparison corrections to the univariate analyses. However, the results are interpreted with caution, considering their effect sizes, confidence intervals, and subsequent validation within the structural equation model.

Following the initial fit of the theoretical model, a refinement process was undertaken to enhance parsimony and goodness-of-fit. This process was guided by two primary criteria:

1. Theory-driven: theoretical plausibility was prioritized. Paths with high Modification Indices (MIs) were not added if they lacked theoretical support.
2. Data-driven: for theoretically permissible modifications, paths with MIs > 10 were considered. Concurrently, a stepwise elimination approach was employed, sequentially removing paths that were statistically non-significant (*p* ≥ 0.05), re-fitting the model after each removal, and observing changes in fit indices.

The specific refinement process was as follows: The initial model contained all 17 theoretically hypothesized paths (H1-H17). Based on the criteria above, paths H3 (Internet addiction → Depression), H4 (Perceived social support → Depression), H5 (Parental attachment → Depression), H10 (Childhood abuse → Perceived social support), H11 (Childhood abuse → Parental attachment), H12 (Childhood abuse → Perceived stress), H16 (Perceived social support → Perceived stress), and H17 (Parental attachment → Perceived stress) were sequentially removed due to *p*-values > 0.05. Furthermore, the modification indices suggested that adding a path from ‘Negative coping’ to ‘Internet addiction’ (MI > 15) could significantly improve model fit. As this path was theoretically justifiable (negative coping tendencies might predispose individuals to seek escape online), it was added. The final, simplified model represents the best-fitting model reported in the manuscript.

### 2.7 Ethics

Prior to the initiation of this study, the study protocol, informed consent form, and related documents were submitted to the Ethics Review Committee of a university in Sichuan for review and were approved. During the screening phase prior to participant involvement, the research team will provide detailed explanations of the study’s purpose, procedures, potential risks, and benefits. Written informed consent will be obtained only after ensuring participants fully understand the information and voluntarily agree to participate.

## 3 Results

### 3.1 Questionnaire response

1,355 questionnaires were distributed at baseline, with 1,284 returned (response rate: 94.76%). After excluding invalid responses, 1,227 valid samples remained. Baseline depression cases were excluded, leaving 988 non-depressed participants for follow-up.

Of these, 917 were successfully followed up. After excluding invalid responses, 880 samples (95.97%) were included: 474 males (53.86%) and 406 females (46.14%). The depression group comprised 154 individuals (17.50%), and the non-depression group 726 (82.50%).

### 3.2 Between-group comparisons

#### 3.2.1 Comparison of continuous variables between groups

(1) Protective factors: the depression group had lower scores for self-esteem, positive coping, perceived social support, father attachment, mother attachment, and parental attachment compared to the non-depression group.
(2) Risk factors: the depression group had higher scores for all childhood abuse dimensions (except sexual abuse), negative life events, total stress load, perceived stress, internet addiction, and negative coping. Detailed results are shown in Table 2.

**Table 2:** Comparison of continuous variables between groups.

Variable	Non-depression group		Depression group		*p-*value
	Median	IQR	Median	IQR	
Past experience factors					
Childhood emotional abuse	5.00	1.00	7.00	3.75	<0.001
Childhood physical abuse	5.00	0.00	5.00	2.00	<0.001
Childhood sexual abuse	5.00	0.00	5.00	0.00	0.001
Childhood emotional neglect	7.00	4.00	11.00	7.00	<0.001
Childhood physical neglect	5.00	3.00	8.00	4.00	<0.001
Total childhood abuse score	30.00	7.00	37.00	13.00	<0.001
Personality factors					
Self-esteem	32.00	8.00	28.00	5.00	<0.001
Interpersonal factors					
Perceived social support	14.00	4.00	12.00	5.00	<0.001
Parental attachment	82.00	17.00	62.00	16.00	<0.001
Maternal attachment	41.50	8.00	32.00	10.75	<0.001
Paternal attachment	41.00	10.00	30.50	8.75	<0.001
Stress factors					
Interpersonal negative events	0.00	2.00	2.00	4.00	<0.001
Academic negative events	2.00	3.00	4.00	4.00	<0.001
Punishment events	0.00	0.00	0.00	3.00	<0.001
Loss events	0.00	2.00	2.00	5.75	<0.001
Adaptation events	1.00	2.00	2.00	5.00	<0.001
Total stress load (impact score)	6.00	8.00	14.00	17.75	<0.001
Total number of stressful events	7.00	7.00	11.00	12.00	<0.001
Perceived stress	36.50	11.00	42.00	4.00	<0.001
Feelings of uncontrollable stress	20.00	6.00	26.00	6.00	<0.001
Feelings of tension	16.00	5.00	18.00	8.00	0.007
Cognitive factors					
Positive coping	35.00	7.00	29.50	20.00	<0.001
Negative coping	26.00	8.00	30.00	7.75	<0.001
Other factors					
Internet addiction	29.00	12.75	34.00	20.00	<0.001

#### 3.2.2 Comparison of categorical variables between groups

A significantly higher prevalence of depression was observed among non-only children compared to only children (21.37% vs. 14.76%, *p* = 0.014), and among students holding rural household registration versus urban registration (22.22% vs. 15.88%, *p* = 0.039). Participants identifying as homosexual or bisexual reported markedly higher depression rates than their heterosexual peers (32.10% vs. 16.02%, *p* = 0.001). Those with a history of school bullying exhibited significantly greater depression prevalence than those without such experiences (32.79% vs. 16.36%, *p* = 0.002).

Parental education below the high school level was associated with elevated depression rates in both paternal (22.94% vs. 14.29%, *p* = 0.002) and maternal (20.68% vs. 15.06%, *p* = 0.037) education comparisons. Students with left-behind experiences showed higher depression prevalence than those without (21.93% vs. 14.68%, *p* = 0.008). Internet addiction was strongly correlated with depression, with affected individuals showing higher rates than non-affected peers (29.67% vs. 14.33%, *p* < 0.001). A clear dose–response relationship emerged in internet addiction severity, with mild, moderate, and severe addiction groups demonstrating progressively higher depression rates (25.00, 60.00, and 100.00%, respectively) compared to the non-addicted group (14.33%, *p* < 0.001).

- No statistically significant differences in depression prevalence were found regarding gender, ethnicity, geographic origin, academic major, romantic relationship status, or parental history of mental illness. Detailed results are shown in Table 3.
- The univariate analysis results (Table 3) revealed that the detection rate of depression was significantly higher among non-only children compared to only children (21.37% vs. 14.76%, *p* = 0.014), and students with rural household registration exhibited a higher risk of depression than their urban counterparts (22.22% vs. 15.88%, *p* = 0.039). It is particularly noteworthy that the depression detection rate among sexual minority (LGBTQ+) individuals was as high as 32.10%, which was significantly greater than that among heterosexual students (16.02%, *p* = 0.001). This substantial disparity highlights the necessity and urgency of providing targeted mental health support for sexual minority groups on campus.

**Table 3:** Comparison of categorical variables between groups.

Variable	Non-depression group		Depression group		*p-*value
	*N*	*n*(%)	*N*	*n*(%)	
Gender					
Male	384	81.01	90	18.99	0.214
Female	342	81.29	64	18.71	
Ethnicity					
Han	641	83.03	131	16.97	0.330
Ethnic minority	85	78.70	23	21.30	
Only child					
Yes	439	85.24	76	14.76	0.014
No	287	78.63	78	21.37	
Household registration (Hukou) type					
Urban	551	84.12	104	15.88	0.039
Rural	175	77.78	50	22.22	
Region of origin					
North China	127	81.41	29	18.59	0.944
East China	69	85.19	12	14.81	
West China	292	81.79	65	18.21	
South China	163	83.16	33	16.84	
Central China	75	83.33	15	16.67	
Major/category of study					
Sciences	188	83.93	36	16.07	0.897
Engineering	195	82.28	42	17.72	
Humanities	138	83.12	28	16.88	
Medical sciences	208	82.21	45	17.79	
Relationship status					
In a relationship	119	81.51	27	18.49	0.821
Single	607	82.70	127	17.30	
Sexual orientation					
Heterosexual	671	83.98	128	16.02	0.001
Non-heterosexual (LGB)	55	67.90	26	32.10	
Experience of campus bullying					
No	685	83.64	134	16.36	0.002
Yes	41	67.21	20	32.79	
Father’s education level					
High school or below	252	77.06	75	22.94	0.002
Above high school	474	85.71	79	14.29	
Mother’s education level					
High school or below	303	79.32	79	20.68	0.037
Above high school	423	84.94	75	15.06	
Parental history of mental illness					
Yes	6	60.00	4	40.00	0.080*
No	720	82.76	150	17.24	
History of being a “left-behind” child					
No	459	85.32	79	14.68	0.008
Yes	267	78.07	75	21.93	
Internet addiction status					
No	598	85.67	100	14.33	<0.001
Yes	128	70.33	54	29.67	
Internet addiction severity					
None	598	85.67	100	14.33	<0.001*
Mild	120	75.00	40	25.00	
Moderate	8	40.00	12	60.00	
Severe	0	0.00	2	100.00	

*Calculated using Fisher’s exact test.

### 3.3 Structural equation modeling (multivariate analysis)

#### 3.3.1 Model construction

Based on univariate analysis, eight predictive factors were included: childhood abuse, perceived stress, self-esteem, negative coping, perceived social support, parental attachment, and internet addiction.

(1) Direct effects

*H1:* Self-esteem has a direct negative effect on depression.

*H2:* Negative coping has a direct positive effect on depression.

*H3:* Internet addiction has a direct positive effect on depression.

*H4:* Perceived social support has a direct negative effect on depression.

*H5:* Parental attachment has a direct negative effect on depression.

*H6:* Perceived stress has a direct positive effect on depression.

*H7:* Childhood abuse has a direct negative effect on self-esteem.

*H8:* Childhood abuse has a direct positive effect on negative coping.

*H9:* Childhood abuse has a direct positive effect on internet addiction.

*H10:* Childhood abuse has a direct negative effect on perceived social support.

*H11:* Childhood abuse has a direct negative effect on parental attachment.

*H12:* Childhood abuse has a direct positive effect on perceived stress.

*H13:* Self-esteem has a direct negative effect on perceived stress.

*H14:* Negative coping has a direct positive effect on perceived stress.

*H15:* Internet addiction has a direct positive effect on perceived stress.

*H16:* Perceived social support has a direct negative effect on perceived stress.

*H17:* Parental attachment has a direct negative effect on perceived stress.

(2) Indirect effects

*H18:* Childhood abuse has an indirect positive effect on depression through multiple mediators.

*H19:* Self-esteem has an indirect negative effect on depression through stress mediation.

*H20:* Negative coping has an indirect positive effect on depression via stress pathways.

*H21:* Internet addiction has an indirect positive effect on depression through stress mediation.

*H22:* Perceived social support has an indirect negative effect on depression via stress reduction.

*H23:* Parental attachment has an indirect negative effect on depression through stress regulation.

**Figure 1:**
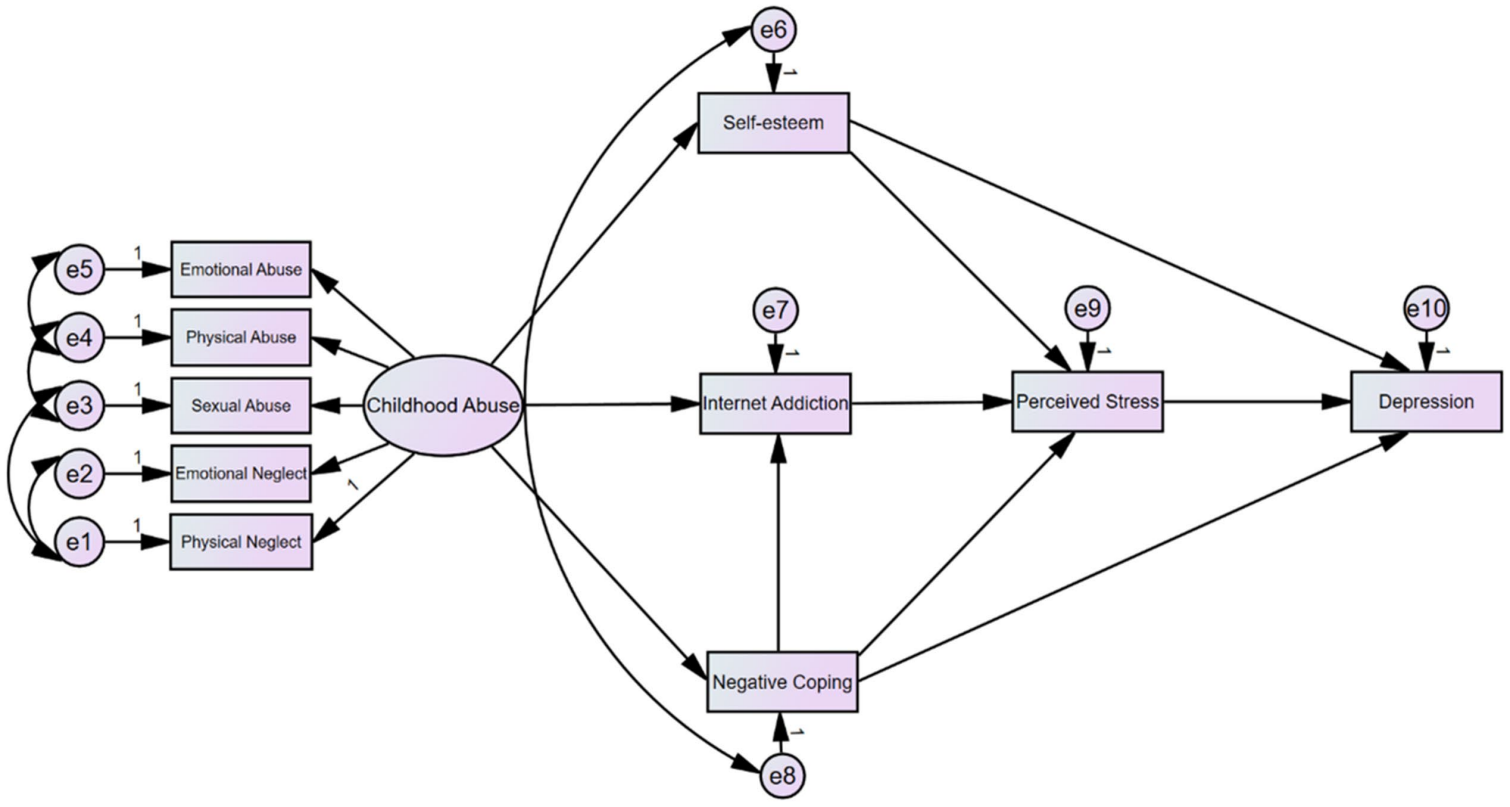
Structural equation model diagram.

Based on a literature review and the results of univariate analysis, this study initially proposed a theoretical hypothesis comprising 23 paths (H1-H23). Following model identification and goodness-of-fit tests, we employed a stepwise modification method, removing statistically non-significant paths (H3, H4, H5, H10, H11, H12, H16, H17, H22, H23) and adding new paths supported by the data (e.g., negative coping → internet addiction). The final model (Figure 1) achieved excellent fit indices (NFI = 0.951, CFI = 0.959, RMSEA = 0.071), indicating that the model fits the actual data well.

The final model is shown in Figure 1. The final structural equation model (Figure 1) reveals a chain mediation mechanism. Childhood abuse, as a distal risk factor, first exerts negative effects on an individual’s psychological resources (self-esteem) and behavioral patterns (negative coping, internet addiction). These factors subsequently collectively influence the individual’s level of perceived stress, with the buffering effect of self-esteem (*β* = −0.54) and the exacerbating effect of negative coping (β = 0.43) being particularly critical. Ultimately, perceived stress emerges as the most direct proximal predictor of depression (β = 0.57). The model diagram intuitively illustrates the complete “causal chain” from early risk factors to recent onset of depression, clarifying the hierarchical relationships and interactive mechanisms among childhood trauma, self-esteem, coping styles, internet use behaviors, and perceived stress in the pathogenesis of depression.

The adaptation status of the final model is presented in Table 4. The final model demonstrated a chi-square/degrees of freedom ratio of 5.448, with all fit indices (NFI, CFI, IFI, GFI, AGFI, RMSEA) meeting established standards, indicating relatively ideal model fit.

**Table 4:** Final model adaptation indices.

Goodness-of-fit indices	NFI	CFI	IFI	GFI	AGFI	RMSEA
Model fit indices	>0.9	>0.9	>0.9	>0.9	>0.9	<0.08
Fit statistics	0.951	0.959	0.960	0.971	0.936	0.071

The final structural equation model path results are systematically presented in Table 1, revealing the complex network of direct, indirect, and total effects among key variables influencing depression among college students.

#### 3.3.2 Results analysis of model

##### 3.3.2.1 Direct effects

The interpretation of the structural equation model path results that follows is based on the unstandardized coefficients reported in Table 1. For instance, the total effect of childhood abuse on depression is 3.22, indicating that, with all other variables held constant, for each one-unit increase in the total childhood abuse score, the depression score is expected to increase by 3.22 units.

(1) Direct effects on depression: ① Self-esteem demonstrated a significant negative direct effect on depression (*β* = −0.57, 95% CI: −0.70 to −0.45), indicating that students with higher baseline self-esteem exhibited lower depression levels at follow-up, confirming its protective role against depressive symptoms.② Negative coping style showed a significant positive direct effect (*β* = 0.15, 95% CI: 0.06 to 0.24), suggesting that students employing more negative coping strategies at enrollment were more likely to develop depression during the follow-up period. ③ Perceived stress exhibited the strongest positive direct effect (*β* = 0.57, 95% CI: 0.48 to 0.66), indicating that higher stress levels during the college adaptation period significantly predicted increased depression severity. Among all direct pathways to depression, perceived stress emerged as the most influential predictor.
(2) Inter-factor direct effects: ① Childhood abuse significantly negatively predicted self-esteem (*β* = −2.34, 95% CI: −2.89 to −1.78), demonstrating that greater childhood trauma exposure correlated with lower self-worth. ② Childhood abuse positively predicted negative coping (β = 2.66, 95% CI: 1.94 to 3.37), revealing its role in fostering maladaptive coping theorized mediating pathways. ③ Childhood abuse directly contributed to internet addiction (*β* = 2.70, 95% CI: 1.57 to 3.82), establishing a pathway from early trauma to compulsive internet use. ④ Self-esteem negatively influenced perceived stress (β = −0.54, 95% CI: −0.63 to −0.46), showing that students with lower self-esteem experienced greater stress during college transition.⑤ Negative coping amplified perceived stress (*β* = 0.43, 95% CI: 0.37 to 0.48), indicating that ineffective coping strategies exacerbated stress perception. ⑥ Internet addiction positively affected perceived stress (*β* = 0.06, 95% CI: 0.03 to 0.09), suggesting that problematic internet use contributed to heightened stress levels.

##### 3.3.2.2 Indirect effects

(1) The total effect of childhood abuse on depression is as high as 3.22 (95% CI: 2.34–4.99) (unstandardized coefficient), which carries clear clinical significance. Childhood abuse exhibits the largest total effect on depression (β = 3.22, 95% CI: 2.34–4.99), confirming that early negative experiences are the strongest predictor of depression among college students. This influence is realized through multiple mediating pathways: on one hand, childhood abuse significantly reduces individuals’ self-esteem levels (β = −2.34, 95% CI: −2.89 to −1.78); on the other hand, it significantly exacerbates tendencies toward negative coping (*β* = 2.66, 95% CI: 1.94–3.37) and internet addiction behaviors (β = 2.70, 95% CI: 1.57–3.82). The relatively narrow confidence intervals of these path coefficients also indicate the precision of the estimates, collectively delineating a clear pathway through which childhood trauma impacts psychological health. Taking the commonly used cutoff score (53 points) of the Self-Rating Depression Scale (SDS) as a reference, this effect size suggests that a significant increase in the childhood abuse score may directly lead an individual to cross from the normal range to near the clinical threshold of mild or even moderate depression. This strongly indicates that early trauma intervention should become an absolute priority in university-based depression prevention efforts. (2) Self-esteem showed significant negative indirect effects on depression (*β* = −0.31, 95% CI: −0.39 to −0.24), primarily mediated through stress reduction pathways. (3) Negative coping demonstrated positive indirect effects (β = 0.26, 95% CI: 0.20 to 0.31) by amplifying perceived stress and reinforcing internet addiction behaviors. (4) Internet addiction contributed to depression through stress mediation (β = 0.03, 95% CI: 0.02 to 0.06), indicating its role in the psychopathological cascade. Although the indirect effect of internet addiction on depression through perceived stress reached statistical significance, its effect size was relatively small (*β* = 0.03). This suggests that, within the multifactorial model constructed in this study, internet addiction may primarily function as a “downstream manifestation” or an “exacerbating factor” of other core risk factors (such as childhood trauma and negative coping). Its direct harm independent of the stress pathway appears to be relatively limited. This finding indicates that interventions targeting internet addiction should be integrated with addressing its underlying psychological drivers (such as trauma recovery and coping skills training) to achieve more effective outcomes, rather than focusing solely on abstinence from internet use.

##### 3.3.2.3 Total effects analysis

Path coefficients revealed that childhood abuse exerted the strongest total effect on depression (β = 3.22), followed by self-esteem (β = −0.88), perceived stress (β = 0.57), negative coping (β = 0.41), and internet addiction (β = 0.03). This hierarchy highlights childhood trauma as the primary distal risk factor, while underscoring the central mediating role of perceived stress in the depression development pathway among college students.

The comprehensive model demonstrates that psychological factors operate through both direct and indirect pathways, with perceived stress serving as a critical hub connecting early adverse experiences to depressive outcomes. These findings emphasize the importance of addressing stress perception and self-esteem in preventive interventions targeting at-risk college populations.

It is noteworthy that the initially hypothesized direct protective pathways of “perceived social support” and “parental attachment” on depression (H4, H5) were not statistically supported in the final model. This may suggest that, for freshmen who have entered the university environment and are separated from their families, the protective effects of early family relationships (parental attachment) and generalized perceived social support may operate more through buffering perceived stress (i.e., the non-significant pathways H16, H17 in the model) or by influencing other mediating variables (such as self-esteem), rather than directly impacting depressive symptoms. This finding prompts us to reconsider that, for this specific group of university freshmen, building immediate and effective new social support systems on campus (such as peer counseling and mentorship programs) may be just as important as tracing back to their early family resources.

## 4 Discussion

This study employs a longitudinal design and structural equation modeling to systematically elucidate the multidimensional mechanisms of depression among college students, establishing an integrative framework that incorporates psychological traits, early-life experiences, and behavioral patterns. The results reveal that self-esteem influences depressive symptoms through dual pathways: High self-esteem indirectly reduces an individual’s vulnerability to depression primarily through the pathway of enhancing psychological resilience (indirect effect β ≈ −0.31, *p* < 0.001), while low self-esteem indirectly exacerbates depressive symptoms through increased perceived stress, accounting for 38.7% of the total indirect effect. These findings are consistent with the diathesis-stress model and prior research on self-esteem and depression [e.g., (31–38)].

Furthermore, childhood abuse contributes to depression via three mediating theorized mediating pathways: it reduces self-esteem (β = −0.19, *p* < 0.01), reinforces negative coping tendencies (β = 0.14, *p* < 0.05), and promotes internet addiction (β = 0.08, *p* < 0.05), which in turn exacerbates social withdrawal and perceived stress. These pathways align with existing literature on trauma and maladaptive behavior [e.g., (39)]. Notably, internet addiction was found to positively affect depression through perceived stress. This supports the “Escape from Reality Self-Regulation Theory,” which posits that individuals may immerse themselves in the internet to escape negative emotions arising from childhood trauma or real-life stress. However, in the long term, this avoidance behavior tends to exacerbate emotional disorders. Additionally, the prevalence of depression among sexual minority (LGBTQ+) students was significantly higher than among heterosexual students (32.10% vs. 16.02%, *p* < 0.001), underscoring the need for targeted mental health support for this population.

The results of this study not only support the classic “Diathesis-Stress” theoretical framework but, more importantly, refine the connotation of “diathesis” and clarify the specific psychological mechanisms through which it operates on “stress.” The model reveals that childhood abuse, as a distal diathesis, primarily shapes three proximal psychological and behavioral traits—low self-esteem, negative coping, and a tendency toward internet addiction—which in turn significantly amplify the perceived stress (stressor) faced by individuals during the university adaptation period, ultimately leading to depression. This chain of pathways operationalizes the abstract concept of “diathesis” into measurable psychological and social factors and clearly delineates the complete process from early risk to recent onset, providing an important empirical supplement and mechanistic elaboration to the theoretical model (40, 41).

Based on the above findings, we propose the following more targeted, evidence-based, multi-tiered intervention strategies:

1. Primary prevention (precision screening and universal promotion): during freshman mental health screenings, priority should be given to identifying students with high childhood trauma exposure, low self-esteem, high negative coping tendencies, and high risk of internet use for targeted attention. Universal mental health education should focus on teaching stress management techniques and positive coping strategies, closely aligned with the common adaptation challenges faced by freshmen (42).
2. Secondary intervention (targeting specific risk pathways):
  - Addressing the childhood trauma pathway: provide school-based Trauma-Focused Cognitive Behavioral Therapy (TF-CBT) group sessions for students with significant childhood trauma experiences, aiming to repair negative self-schemas rather than offering only general support.
  - Addressing the self-esteem and coping pathway: promote self-esteem enhancement workshops and Problem-Solving Skills Training (PSST) to help students build positive self-evaluation and effective stress coping methods, thereby reducing their perceived stress levels.
  - Addressing the internet use pathway: implement rational internet use education, emphasizing the harms of using the internet as an emotional escape tool and guiding students to develop offline hobbies and social activities (43).
3. Tertiary Intervention (high-risk group correction): for students already exhibiting internet addiction or mild depressive symptoms, provide timely individual psychological counseling and necessary referrals. The intervention should simultaneously address the addictive behavior and the underlying emotional issues and psychological needs (44).

These intervention targets directly correspond to the key mechanistic pathways identified in this study, forming a complete logical chain from “risk identification” to “core mechanism intervention”.

Consequently, institutional mental health initiatives must evolve from isolated interventions toward integrated, systemic support. We propose the establishment of a multi-tiered “screening–prevention–intervention” framework. This includes (1) implementing routine psychological screening to accurately identify high-risk individuals with histories of childhood trauma; (2) delivering universal mental health education focused on strengthening self-esteem and fostering adaptive stress management skills; and (3) providing specialized psychological counseling and trauma-informed interventions for high-risk students. A coordinated, multi-level strategy is essential to effectively mitigate depressive risk and enhance overall population mental health on college campuses.

## 5 Limitations and future directions

A key limitation of this study concerns causal inference. Although the prospective design and the exclusion of baseline depression cases strengthen the temporal ordering of variables, all predictor variables were measured only at baseline (T1). This precludes an analysis of how dynamic changes in variables like coping styles or perceived stress during the three-month follow-up period might influence the onset of depression. Furthermore, the SEM analysis establishes association but cannot rule out the influence of unmeasured confounders. Therefore, our findings primarily support robust predictive relationships and plausible mediating pathways. Future research should incorporate more waves of data collection and utilize analytical methods such as Cross-Lagged Panel Models (CLPM) or Random-Intercept Cross-Lagged Panel Models (RI-CLPM) to more rigorously examine the potential causal dynamics and bidirectional effects among these psychological constructs over time.

## Data Availability

The original contributions presented in the study are included in the article/supplementary material, further inquiries can be directed to the corresponding author.
